# Co-inhibition of PGF and VEGF blocks their expression in mononuclear phagocytes and limits neovascularization and leakage in the murine retina

**DOI:** 10.1186/s12974-019-1419-2

**Published:** 2019-02-07

**Authors:** Carsten Balser, Anne Wolf, Marc Herb, Thomas Langmann

**Affiliations:** 1 Laboratory for Experimental Immunology of the Eye, Department of Ophthalmology, University of Cologne, Faculty of Medicine and University Hospital Cologne, 50931 Cologne, Germany; 2 Institute for Medical Microbiology, Immunology and Hygiene, 50931 Cologne, Germany; 3 Center for Molecular Medicine Cologne (CMMC), University of Cologne, 50931 Cologne, Germany

**Keywords:** Age-related macular degeneration, Neovascular AMD, Choroidal neovascularization, Retinal degeneration, Microglia, Aflibercept, Vascular endothelial growth factor (VEGF), Placental growth factor (PGF), Laser CNV

## Abstract

**Background:**

Age-related macular degeneration (AMD) is a leading cause of visual impairment in the elderly. The neovascular (wet) form of AMD can be treated with intravitreal injections of different anti-vascular endothelial growth factor (VEGF) agents. Placental growth factor (PGF) is another member of the VEGF family of cytokines with pro-angiogenic and pro-inflammatory effects. Here, we aimed to compare single and combined inhibition of VEGF-A and PGF in the laser-induced mouse model of choroidal neovascularization (CNV) with a focus on the effects on retinal mononuclear phagocytes.

**Methods:**

CNV was induced in C57BL/6J mice using a YAG-Laser. Immediately after laser damage antibodies against VEGF-A (aVEGF), anti-PGF (aPGF), aVEGF combined with aPGF, aflibercept, or IgG control were injected intravitreally in both eyes. Three and 7 days after laser damage, the vascular leakage was determined by fluorescence angiography. Lectin staining of retinal and RPE/choroidal flat mounts was used to monitor CNV. In situ mRNA co-expression of Iba1, VEGF and PGF were quantified using in situ hybridization. Retinal and RPE/choroidal protein levels of VEGF and PGF as well as the pro-inflammatory cytokines IL-6, IL1-beta, and TNF were determined by ELISA.

**Results:**

Early (day 3) and intermediate (day 7) vascular leakage and CNV were significantly inhibited by PGF and VEGF-A co-inhibition, most effectively with the trap molecule aflibercept. While VEGF-A blockage alone had no effects, trapping PGF especially with aflibercept prevented the accumulation of reactive microglia and macrophages in laser lesions. The lesion-related mRNA expression and secretion of VEGF-A and PGF by mononuclear phagocytes were potently suppressed by PGF and partially by VEGF-A inhibition. Protein levels of IL-6 and IL1-beta were strongly reduced in all treatment groups.

**Conclusions:**

Retinal inhibition of PGF in combination with VEGF-A prevents vascular leakage and CNV possibly via modulating their own expression in mononuclear phagocytes. PGF-related, optimized strategies to target inflammation-mediated angiogenesis may help to increase efficacy and reduce non-responders in the treatment of wet AMD patients.

**Electronic supplementary material:**

The online version of this article (10.1186/s12974-019-1419-2) contains supplementary material, which is available to authorized users.

## Background

Age-related macular degeneration (AMD) is a frequent cause of visual impairment in the Western world, leading to considerable limitations in the daily life of elderly people [1]. There is no treatment available for dry AMD, and the only approved therapy for neovascular AMD is effective in inhibiting the vascular endothelial growth factor (VEGF) [2]. The fact that patients are refractory to anti-VEGF (aVEGF) treatments and that adverse events may occur underlines the need for new therapeutic strategies [3].

The pathogenesis of AMD is primarily characterized by a loss of retinal pigment epithelium (RPE) function. As a consequence, progressive degeneration of photoreceptors occurs and a strong immunological response is mounted in the retina and RPE/choroid. This latent para-inflammation is characterized by local microgliosis, infiltration of inflammatory macrophages in the subretinal space, and dysregulation of the complement system [4]. In the healthy retina, resident microglia are required for the maintenance of synapses and thereby help to preserve tissue integrity [5]. However, by producing pro-angiogenic cytokines and growth factors including VEGF and placental growth factor (PGF), mononuclear phagocytes may also play a role in neovascularization [6]. Therefore, modulation of the pro-inflammatory state of microglia and macrophages may be a therapeutic strategy for retinal degenerative diseases [7].

There are currently different therapeutic biologics neutralizing ocular VEGF. Bevacizumab and ranibizumab only bind VEGF-A, whereas aflibercept is capable of targeting both, VEGF and PGF dimers [8]. Several studies suggest that PGF, also a member of the VEGF family, plays a crucial role in immune cell-related neovascularization as shown by genetic deletion of PGF in mice [9]. PGF plays a role in several retinal vascular diseases as has been excellently reviewed recently [10]. Thus, increased PGF has been identified in ocular fluids and tissue from patients with diabetic retinopathy [11, 12]), glaucoma [13], and neovascular AMD [14, 15]. High PGF levels are also present in the murine laser model of choroidal neovascularization, and PGF inhibition by either antibodies [16, 17] or trap molecules [18, 19] limits CNV.

Today, there is only limited information which cell types produce PGF in the diseased retina. In diabetic conditions with retinal edema, the RPE has been identified as a possible source for PGF [20, 21]. As there seems to be a direct interplay between PGF, VEGF receptors, and mononuclear phagocytes, these retinal immune cells could also be a potential producer of PGF. Interestingly, PGF but not VEGF triggers chemotaxis of phagocytes in a model of diabetic retinopathy [22]. In other non-retinal inflammatory conditions, PGF potently stimulates monocyte chemotaxis and expression of pro-inflammatory cytokines [23] as well as macrophage survival [24].

In this report, we analyzed the retinal effects of individual and combined intravitreal inhibition of PGF and VEGF using antibodies and the VEGF/PGF trap aflibercept. We specifically focused on the temporal behavior and role of mononuclear phagocytes in the production of PGF and VEGF. We show that microglia and macrophages co-express both angiogenic factors after laser treatment and that aflibercept is highly efficient to limit PGF expression and choroidal neovascularization.

## Methods

### Animals

Experiments were performed with 8–10-week-old C57BL/6J mice of both sexes [20]*.* Animals were housed in an air-conditioned environment with 12-h light-dark schedule and had access to water and food ad libitum. All experimental procedures complied with the German animal welfare law, which is in line with the European Community law, and the ARVO Statement for the Use of Animals in Ophthalmic and Vision Research. The animal protocols used in this study were reviewed and approved by the governmental body responsible for animal welfare in the state of North Rhine-Westphalia, Germany (application no. 81-02.04.2017.A430).

### Laser coagulation

Laser coagulation of the retina was performed by using a slit-lamp-mounted diode laser system by Quantel Medical Vitra (532-nm green laser). For laser treatment, mice were anesthetized by intraperitoneally injecting ketamine hydrochloride (100 mg/kg body weight, Ketavet; Pfizer Animal Health) and xylazine hydrochloride (5 mg/kg body weight, 2% Rompun; Bayer HealthCare) diluted in 0.9% sodium chloride. The pupils of the mice were dilated using phenylephrine 2.5%–tropicamide 0.5% before laser treatment. For fundus fluorescence angiography (FFA), immunohistochemistry (IHC), and in situ hybridization (ISH), three laser burns (energy 125 mW, duration 100 ms, spot size 100 μm) were equally placed around the optic nerve of both eyes [25]. For ELISA measurements of cytokines, the number of laser burns applied per eye was 20. To validate rupture of Bruch’s membrane, post-laser retinal structure and laser lesion size were analyzed in vivo using HRA/OCT. In case of media opacities precluding accurate laser application (pre-existing corneal scar or cataract), insufficient disruption of Bruch’s membrane, or hemorrhages, these eyes were excluded from analyses.

### Drug administration

Animal cages were randomly allocated to the experimental groups. The following compounds (all diluted in 1× PBS) were injected intravitreally immediately after laser pulse application: 1.5 μl of either Aflibercept (10 μg/μl, Eylea, Bayer HealthCare), anti-VEGF-A (5 μg/μl, goat anti-mouse VEGF-AA IgG, AF493-NA, R&D Systems), anti-PGF (5 μg/μl, polyclonal rabbit anti-PGF antibody, ab9542, Abcam), anti-VEGF and anti-PGF combined (each 5 μg/μl), or IgG control (10 μg/μl, normal goat IgG control (AB-108-C, R&D systems). Therefore, a 34-gauge needle was inserted into the vitreous space approximately 1.5 mm below the limbus and the compounds were administered bilaterally with a NanoFil syringe (Word Precision Instruments, Sarasota, FL, USA).

### Fundus fluorescein angiography (FFA)

Vascular leakage was analyzed 3 and 7 days after laser damage. After anesthesia of the animals and dilatation of the pupils, the vascular leakage was determined with the FA-mode of the HRA/OCT device (Spectralis™). One hundred microliters of 2.5% fluorescein (Alcon) diluted in 0.9% sodium chloride were injected intraperitoneally. Late-phase images were taken 10 min after fluorescein administration. The size of laser spots and vascular leakage was determined using the measuring tool of the Heidelberg software. The pixel intensity was quantified in two regions of interest (ROI) within and one ROI outside each laser spot using the program ImageJ. The background pixel intensity was then subtracted from the laser spot values. The data of three laser spots were averaged to obtain the mean laser-induced leakage per eye.

### Preparation of flat mounts, immunohistochemistry, and image analysis

The eyes were enucleated and fixed in 10% neutral buffered formalin (NBF) for 2 h at room temperature. The dissected retinal and RPE/choroidal flat mounts were permeabilized overnight (5% Triton X-100, 5% Tween-20 in PBS). Unspecific antigens were blocked with BLOTTO (1% milk powder, 0.01% Triton X-100 in PBS) for 1 h at room temperature. The flat mounts were subsequently incubated in the primary antibody overnight at 4 °C (1:1000 dilution of Iba1, rabbit polyclonal, 234 003, Synaptic Systems). Flat mounts were then incubated with a 1:1000 dilution of goat anti-rabbit AlexaFluor 488 nm-conjugated secondary antibody (A11008; Life Technologies) for 1 h. In addition, RPE/choroidal flat mounts were incubated with a 1:10 dilution of primary TRITC-conjugated lectin (L5264; Sigma). After washing, retinal and RPE/choroidal flat mounts were mounted on a microscope slide and embedded with fluorescence mounting medium (S3023; DakoCytomation) [25].

Images were taken with a Zeiss Imager M.2 equipped with an ApoTome.2. The total number of Iba1-positive cells was counted for each laser spot. Cellular morphology was analyzed using a grid system to determine the mean number of grid crossing points per cell [25]. The colored pixel intensity in individual image areas of the laser spots was quantified using the Colored Pixel Counter tool for Fiji.

Areas of choroidal neovascularization in RPE/choroidal flat mounts were measured with the spline function of the graphic tool included in the ZEN software (Zeiss). Data were excluded when it came to damages to the CNV lesion during tissue processing or inability to locate a CNV lesion during imaging of an eyecup.

### In situ hybridization (ISH) and image analysis

ISH of RPE/choroidal flat mounts was performed using the Multiplex Fluorescent Reagent Kit v2 (ACD) according to the protocol of Gross-Thebing et al. [26]. The following probes were used: Iba1 (channel C1 or C3), VEGF (channel C3), and PGF (channel C1). Images were taken with a Zeiss Imager M.2 including an ApoTome.2.

The colored pixel intensity in individual image areas of the laser spots was quantified using the Colored Pixel Counter tool for Fiji.

### Quantification of cytokines

The concentration of cytokines in total retinal or RPE lysates were measured by ELISA according to the manufacturer’s instructions (R&D Systems). Absorbance was quantified using a TriStar^2^ multimode plate reader LB 942 (Berthold Technologies).

### Statistical analysis

All data were analyzed using GraphPad PRISM (version 7) using analysis of variance (ANOVA) and Tukey’s post-test, *P < 0.05, **P < 0.01, and ***P ≤ 0.001. The data are shown as mean ± SEM. All analyses were performed after random consecutive numbering of animals, which was only revealed after finishing all analyses.

## Results

### Aflibercept strongly inhibits vascular leakage and choroidal neovascularization

**Fig. 1:**
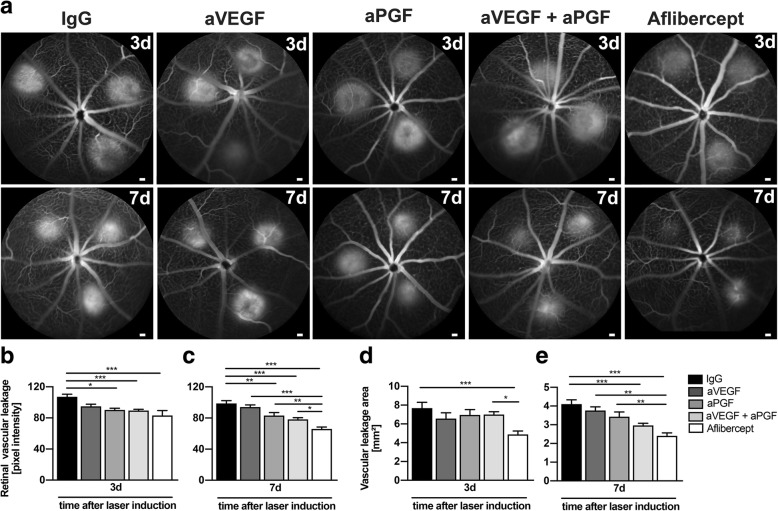
Effects of PGF and VEGF inhibition on vascular leakage in fundus fluorescein angiography. **a** Representative fundus fluorescein angiography images of animals of the indicated treatment groups 3 and 7 days after laser-induced damage. **b** Quantification of vascular leakage by analyzing pixel intensities 3 days after laser-induced damage (*n* = 13 eyes for IgG, *n* = 14 eyes for aVEGF, *n* = 13 eyes for aPGF, *n* = 20 eyes for aVEGF/aPGF, *n* = 13 eyes for aflibercept). **c** Quantification of vascular leakage by analyzing pixel intensities 7 days after laser-induced damage (*n* = 26 eyes for IgG, *n* = 14 eyes for aVEGF, *n* = 14 eyes for aPGF, *n* = 16 eyes for aVEGF/aPGF, *n* = 27 eyes for aflibercept). **d** Quantification of vascular leakage area 3 days after laser-induced damage (*n* = 13 eyes for IgG, *n* = 14 eyes for aVEGF, *n* = 13 eyes for aPGF, *n* = 20 eyes for aVEGF/aPGF, *n* = 13 eyes for aflibercept). **e** Quantification of vascular leakage area 7 days after laser-induced damage (*n* = 26 eyes for IgG, *n* = 14 eyes for aVEGF, *n* = 14 eyes for aPGF, *n* = 16 eyes for aVEGF/aPGF, *n* = 27 eyes for aflibercept). Data are shown as mean ± SEM (*P < 0.05, **P < 0.01, ***P < 0.001)

We first compared the effects of inhibiting VEGF-A alone (aVEGF), PGF alone (aPGF), or in combinations (aVEGF plus aPGF, aflibercept) with IgG sham injections in the laser-induced mouse model of CNV. The inflammation-induced vascular leakage was assessed with fundus fluorescein angiography (FFA) at days 3 and 7 (Fig. 1a). The pixel intensities of leakage lesions (Fig. 1b, c) and their total area (Fig. 1d, e) were quantified, respectively. At 3 days after laser induction, anti-PGF alone (aPGF), the combination of aVEGF with aPGF, and aflibercept significantly reduced the pixel intensity of vascular leakage compared to IgG (Fig. 1b). At 7 days after laser, the reduction in leakage intensity was greater with all injected biologics and aflibercept showed the strongest effect with significant differences to all other conditions (Fig. 1c). When analyzing leakage area after laser damage, aflibercept was the only molecule that showed a significant effect after 3 days (Fig. 1d). Aflibercept was also most effective treatment regarding the leakage area at day 7 (Fig. 1e). Only the combined therapy of aVEGF with aPGF and aflibercept showed significant differences compared to IgG treatment, whereas single administration of either aVEGF or aPGF did not affect leakage at this time point (Fig. 1e).

**Fig. 2:**
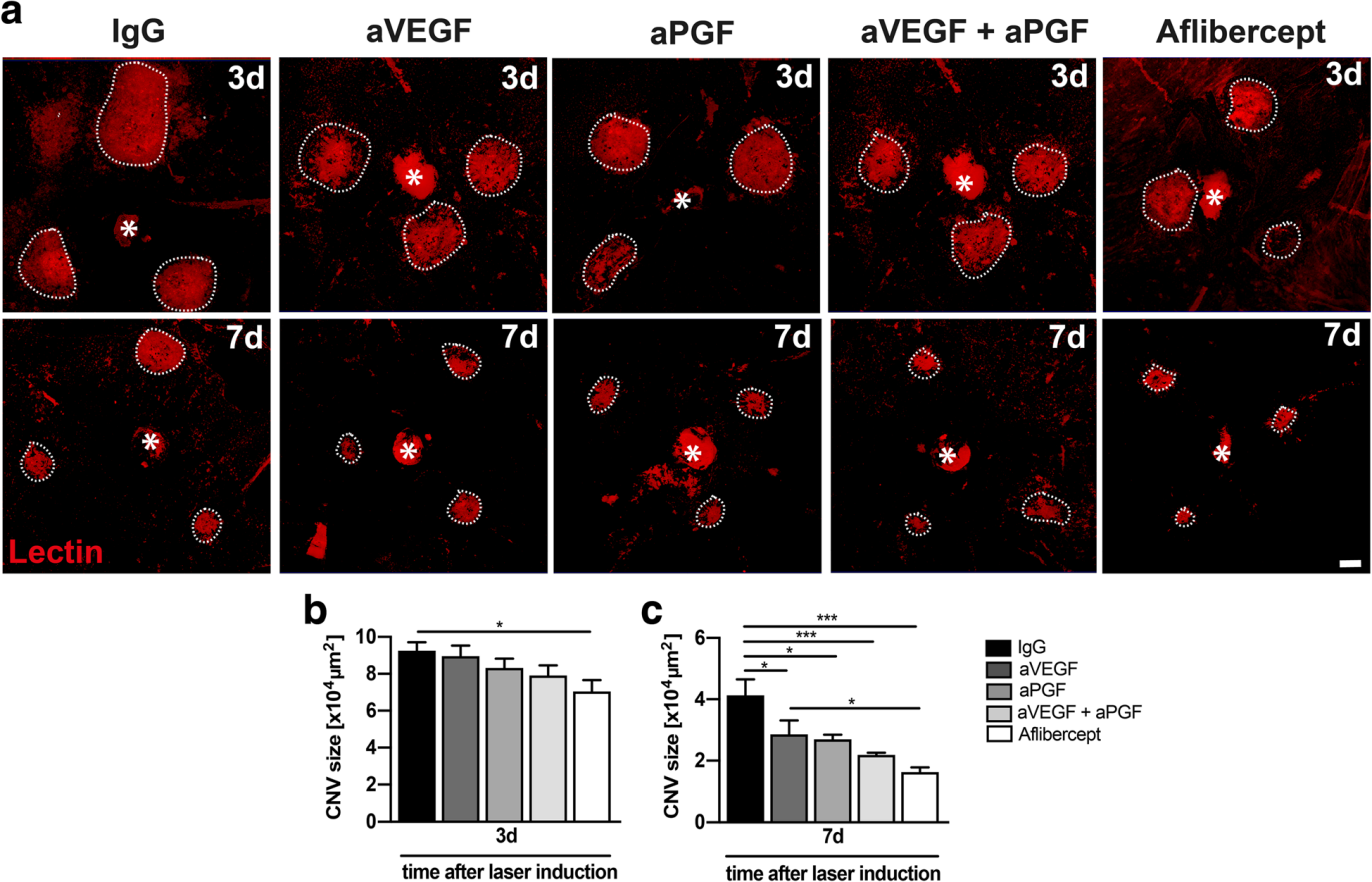
Effects of PGF and VEGF inhibition on choroidal neovascularization. **a** Representative images of lectin-stained RPE/choroidal flat mounts of animals of all treatment groups 3 and 7 days after laser damage. Dashed lines indicate CNV areas, and the asterisks mark the optic nerve head. Scale bare: 100 μm. **b** Quantification of lectin-stained CNV areas in RPE/choroidal flat mounts 3 days after laser coagulation with ZEN software (*n* = 10 eyes for IgG, *n* = 10 eyes for aVEGF, *n* = 11 eyes for aPGF, *n* = 11 eyes for aVEGF/aPGF, *n* = 10 eyes for aflibercept). **c** Quantification of lectin-stained CNV areas in RPE/choroidal flat mounts 7 days after laser coagulation with ZEN software (*n* = 14 eyes for IgG, *n* = 13 eyes for aVEGF, *n* = 14 eyes for aPGF, *n* = 16 eyes for aVEGF/aPGF, *n* = 28 eyes for aflibercept). Data are shown as mean ± SEM (*P < 0.05, **P < 0.01, ***P < 0.001)

We next analyzed neovessel formation by lectin staining of RPE/choroidal flat mounts at days 3 and 7 (Fig. 2). Three days after laser damage only aflibercept reduced CNV compared to the IgG control (Fig. 2b). At day 7, all treatments significantly reduced CNV formation compared to IgG, whereby the combination of aVEGF and aPGF as well as aflibercept led to the strongest decrease in CNV formation (Fig. 2c).

As the laser-CNV model also reflects an inflammation-related wound healing process [27, 28], we analyzed the overall size of the lesions at three different time points after laser (Additional file 1: Figure S1). Over time, the lesion size and the formation of a fibrotic scar decreased in all conditions and no significant effect of any treatment was observed (Additional file 1: Figure S1). This indicates that the applied compounds did not affect wound healing or fibrosis.

### PGF inhibition limits microglia and macrophage responses in retinal and RPE/choroidal flat mounts

**Fig. 3:**
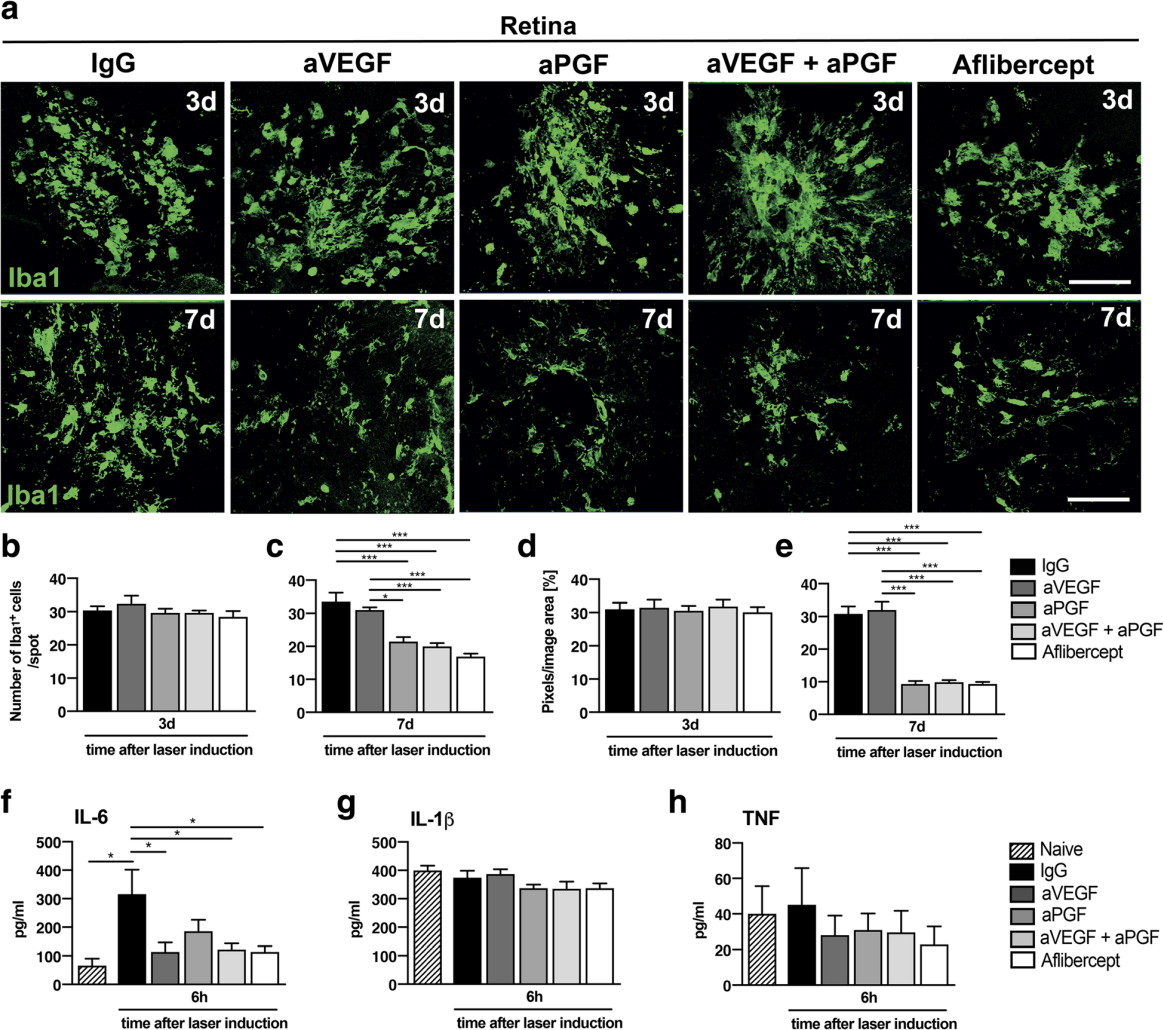
Effects of PGF and VEGF inhibition on microgliosis in retinal flat mounts. **a** Representative images of Iba1-stained microglia/macrophages in single laser spots of retinal flat mounts 3 and 7 days after laser coagulation. Scale bar: 100 μm. **b** Quantification of mononuclear phagocytes per laser spot in retinal flat mounts 3 days after laser-induced damage (*n* = 16 laser spots for IgG, *n* = 14 laser spots for aVEGF, *n* = 14 laser spots for aPGF, *n* = 15 laser spots for aVEGF/aPGF, *n* = 14 laser spots for aflibercept). **c** Quantification of mononuclear phagocytes per laser spot in retinal flat mounts 7 days after laser-induced damage (*n* = 14 laser spots for IgG, *n* = 14 laser spots for aVEGF, *n* = 15 laser spots for aPGF, *n* = 27 laser spots for aVEGF/aPGF, *n* = 28 laser spots for aflibercept). **d** Quantification of Iba1 signals 3 days after laser coagulation in retinal flat mounts by counting the mean of colored pixels per image (*n* = 16 laser spots for IgG, *n* = 14 laser spots for aVEGF, *n* = 14 laser spots for aPGF, *n* = 15 laser spots for aVEGF/aPGF, *n* = 14 laser spots for aflibercept). **e** Quantification of Iba1 signals 7 days after laser coagulation in retinal flat mounts by counting the mean of colored pixels per image (*n* = 14 laser spots for IgG, *n* = 14 laser spots for aVEGF, *n* = 15 laser spots for aPGF, *n* = 27 laser spots for aVEGF/aPGF, *n* = 28 laser spots for aflibercept). **f** Interleukin 6 (IL-6) levels in retinal flat mounts 6 h after laser damage quantified by ELISA (*n* = 8 flat mounts per group). Naive (not lasered) animals were used as controls. **g** Interleukin 1β (IL-1β) levels in retinal flat mounts 6 h after laser damage quantified by ELISA (*n* = 8 flat mounts per group). **h** Tumor necrosis factor (TNF) levels in retinal flat mounts 6 h after laser damage quantified by ELISA (*n* = 8 flat mounts per group). Data are shown as mean ± SEM (*P < 0.05, **P < 0.01, ***P < 0.001)

**Fig. 4:**
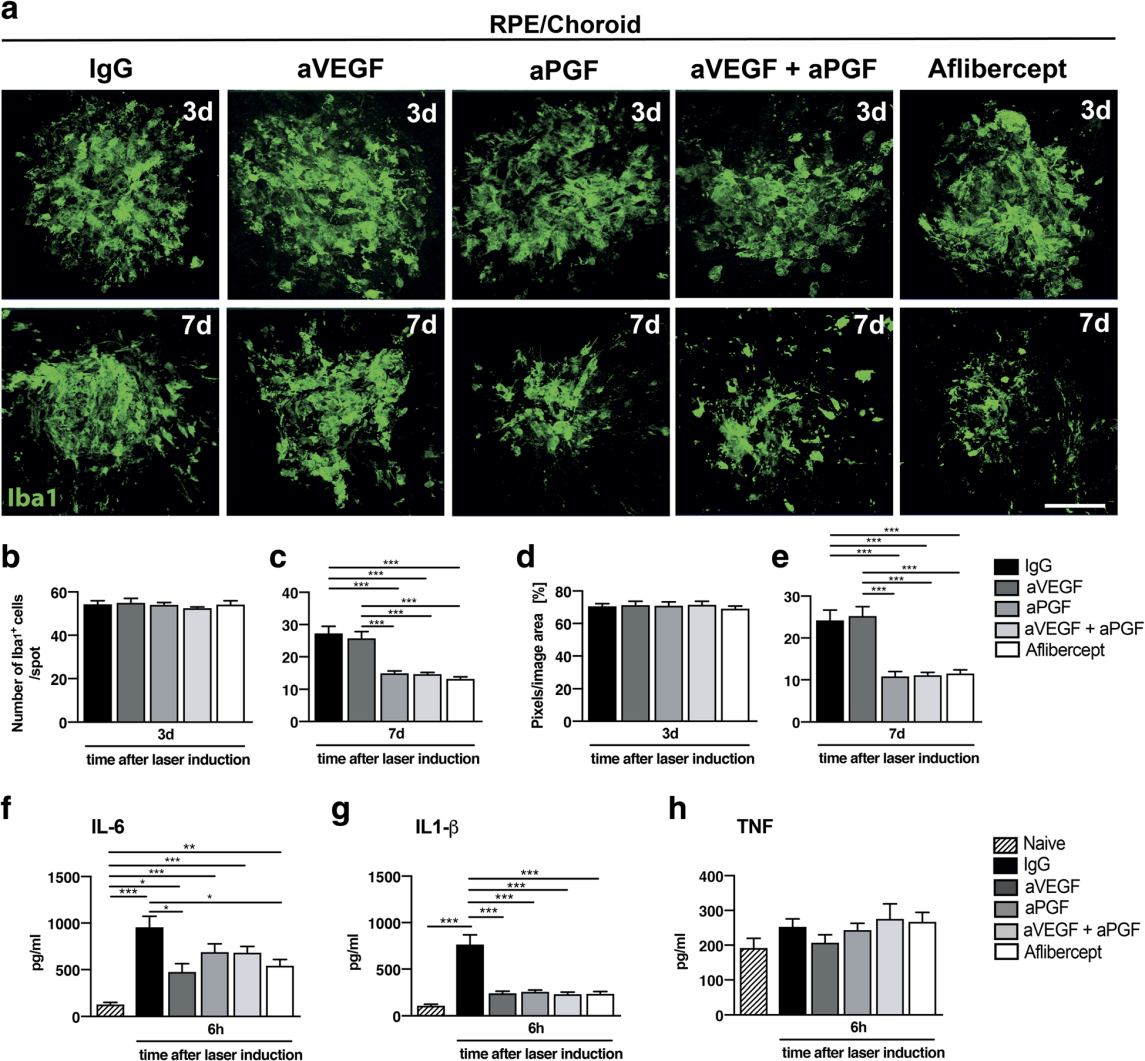
Effects of PGF and VEGF inhibition on mononuclear phagocytes in RPE/choroidal flat mounts. **a** Representative images of Iba1-stained microglia/macrophages in single laser spots of RPE/choroidal flat mounts 3 and 7 days after laser coagulation. Scale bar: 100 μm. **b** Quantification of mononuclear phagocytes per laser spot in retinal flat mounts 3 days after laser-induced damage (*n* = 20 laser spots for IgG, *n* = 10 laser spots for aVEGF, *n* = 12 laser spots for aPGF, *n* = 13 laser spots for aVEGF/aPGF, *n* = 27 laser spots for aflibercept). **c** Quantification of mononuclear phagocytes per laser spot in RPE/choroidal flat mounts 7 days after laser-induced damage (*n* = 11 laser spots for IgG, *n* = 10 laser spots for aVEGF, *n* = 10 laser spots for aPGF, *n* = 15 laser spots for aVEGF/aPGF, *n* = 30 laser spots for aflibercept). **d** Quantification of Iba1 signals 3 days after laser coagulation in RPE/choroidal flat mounts by counting the mean of colored pixels per image (*n* = 20 laser spots for IgG, *n* = 10 laser spots for aVEGF, *n* = 12 laser spots for aPGF, *n* = 13 laser spots for aVEGF/aPGF, *n* = 27 laser spots for aflibercept). **e** Quantification of Iba1 signals 7 days after laser coagulation in RPE/choroidal flat mounts by counting the mean of colored pixels per image (*n* = 11 laser spots for IgG, *n* = 10 laser spots for aVEGF, *n* = 10 laser spots for aPGF, *n* = 15 laser spots for aVEGF/aPGF, *n* = 30 laser spots for aflibercept). **f** Interleukin 6 (IL-6) levels in RPE/choroidal flat mounts 6 h after laser damage quantified by ELISA (*n* = 8 flat mounts per group). Naive (not lasered) animals were used as controls. **g** Interleukin 1β (IL-1β) levels in RPE/choroidal flat mounts 6 h after laser damage quantified by ELISA (*n* = 8 flat mounts per group). **h** Tumor necrosis factor (TNF) levels in RPE/choroidal flat mounts 6 h after laser damage quantified by ELISA (*n* = 8 flat mounts per group). Data are shown as mean ± SEM (*P < 0.05, **P < 0.01, ***P < 0.001)

We next addressed which compound was effective in modulating the activity of Iba1+ microglia and macrophages in the retina (Fig. 3) and the RPE/choroid complex (Fig. 4). Quantification of Iba1^+^ cells per laser spot showed no differences between the treatments 3 days after laser damage (Fig. 3a, b). In contrast, 7 days after laser induction, inhibition of PGF either alone or in combination reduced the number of retinal microglia (Fig. 3c), with the strongest effect after aflibercept injection. These findings were confirmed by quantifying the number of Iba1 signal pixels in each area (Fig. 3d, e).

Since the morphology of microglia often correlates with their function and pro-inflammatory state, we next analyzed their ramification by counting the number of grid cross points per cell. None of the treatments affected the ramification state, indicating that the compounds mainly affected the recruitment of microglia to the laser lesion and not their overall morphology (Additional file 2: Figure S2a, b).

We next focused on the protein levels of three pro-inflammatory cytokines often rapidly produced by reactive microglia [29]. Six hours after laser damage, IL-6 was rapidly secreted in the retinal tissue, whereas IL-1β and TNF did not show laser-induced expression differences (Fig. 3f-h). Treatments with aVEGF, aVEGF combined with aPGF, and aflibercept reduced the secretion of IL-6, while aPGF showed no significant effect (Fig. 3f). Of note, 3 days after laser coagulation, the secretion of IL-6 declined to the level of naive mice (data not shown).

Staining of mononuclear phagocytes in RPE/choroidal flat mounts showed similar results than those in the retina (Fig. 4). Thus, there was no difference between all groups at day 3 (Fig. 4a, b, d), but strong effects at day 7 (Fig. 4c, e). All groups involving PGF inhibition reduced the number of Iba1+ cells in the laser spots at day 7 (Fig. 4c). Again, the analysis of grid crossing points per cell did not reveal any differences for any time point (Additional file 2: Figure S2c,d).

ELISAs of RPE/choroidal flat mounts showed increased IL-6 and IL-1β secretion 6 h after laser damage, while no TNF secretion was detected (Fig. 4f-h). Interestingly, treatment with aflibercept and aVEGF resulted in significantly reduced levels of IL-6 compared to IgG (Fig. 4f), whereas all treatments strongly inhibited IL-1β secretion (Fig. 4g).

### VEGF and PGF inhibition limits their co-expression and secretion in mononuclear phagocytes

**Fig. 5:**
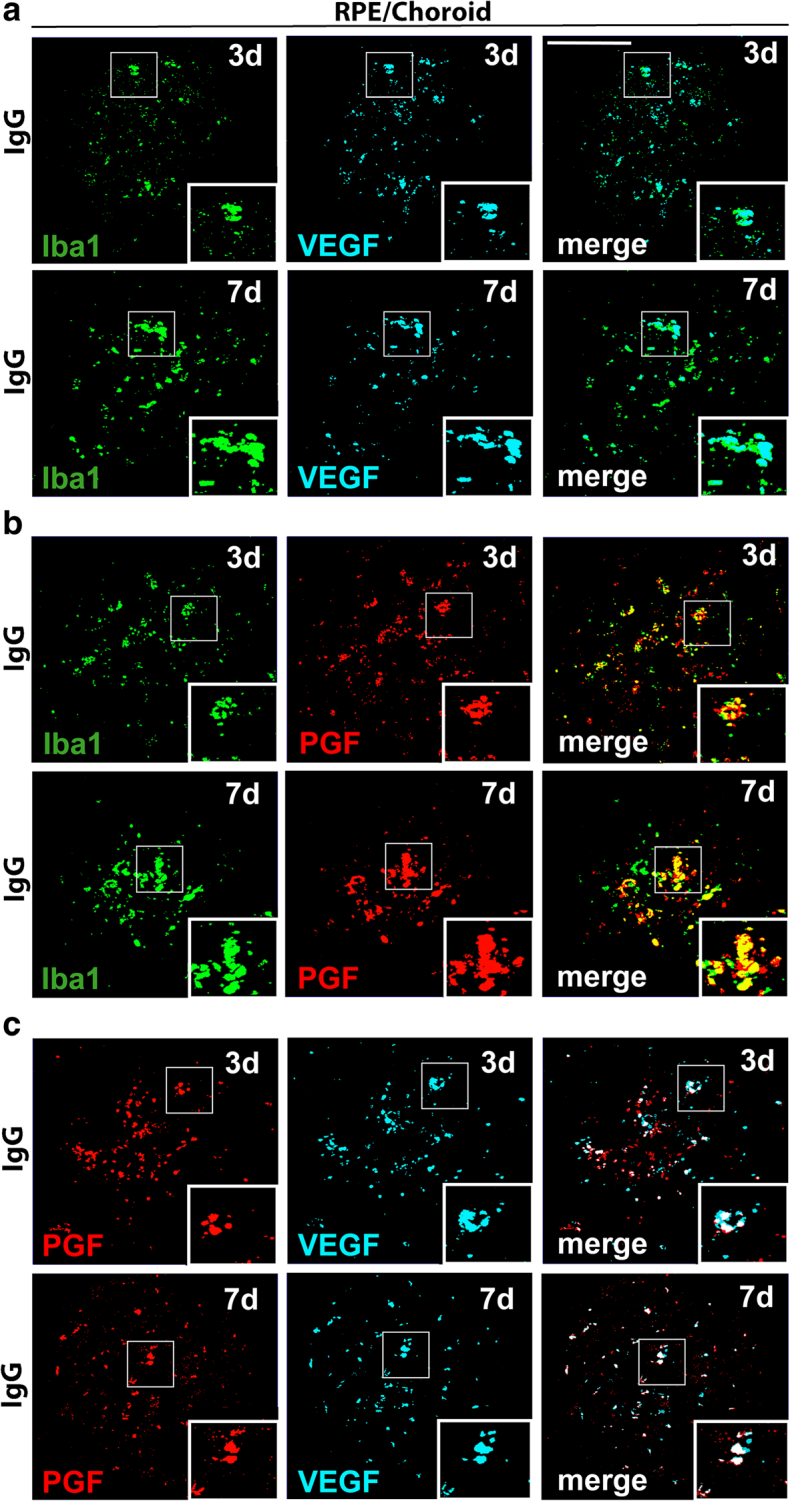
Mononuclear phagocytes co-express PGF and VEGF mRNAs in laser lesions. Scale bar: 100 μm. **a** Representative images of in situ hybridization of single laser spots in RPE/choroidal flat mounts of IgG-treated animals at days 3 and 7. Probes targeted both Iba1 and VEGF. **b** Representative images of in situ hybridization of laser spots in RPE/choroidal flat mounts of IgG-treated animals at days 3 and 7. Probes targeted both Iba1 and PGF. **c** Representative images of in situ hybridization of laser spots in RPE/choroidal flat mounts of IgG-treated animals at days 3 and 7. Probes targeted both PGF and VEGF. The frames show higher magnification areas

Since trapping of PGF significantly attenuated microgliosis and macrophage numbers in the laser lesions, we next asked the question whether these cells themselves actively transcribe VEGF and PGF mRNAs using in situ hybridization (ISH) with specific amplifier probes. We first compared VEGF and PGF mRNA expression together with the marker Iba1. Therefore, ISH with RPE/choroidal flat mounts of lasered mice treated with IgG for 3 and 7 days was performed (Fig. 5a–c). The simultaneous detection of Iba1 (green) and VEGF (blue) showed that most Iba1+ mononuclear phagocytes expressed VEGF in the lesion area (Fig. 5a). Similar results were obtained with PGF (Fig. 5b). Simultaneous hybridization with probes targeting PGF and VEGF showed that the majority of cells, which were positive for VEGF, also expressed PGF at both time points (Fig. 5c*).* These results indicate that Iba1+ cells are capable to co-express significant amounts of VEGF and PGF mRNA.

**Fig. 6:**
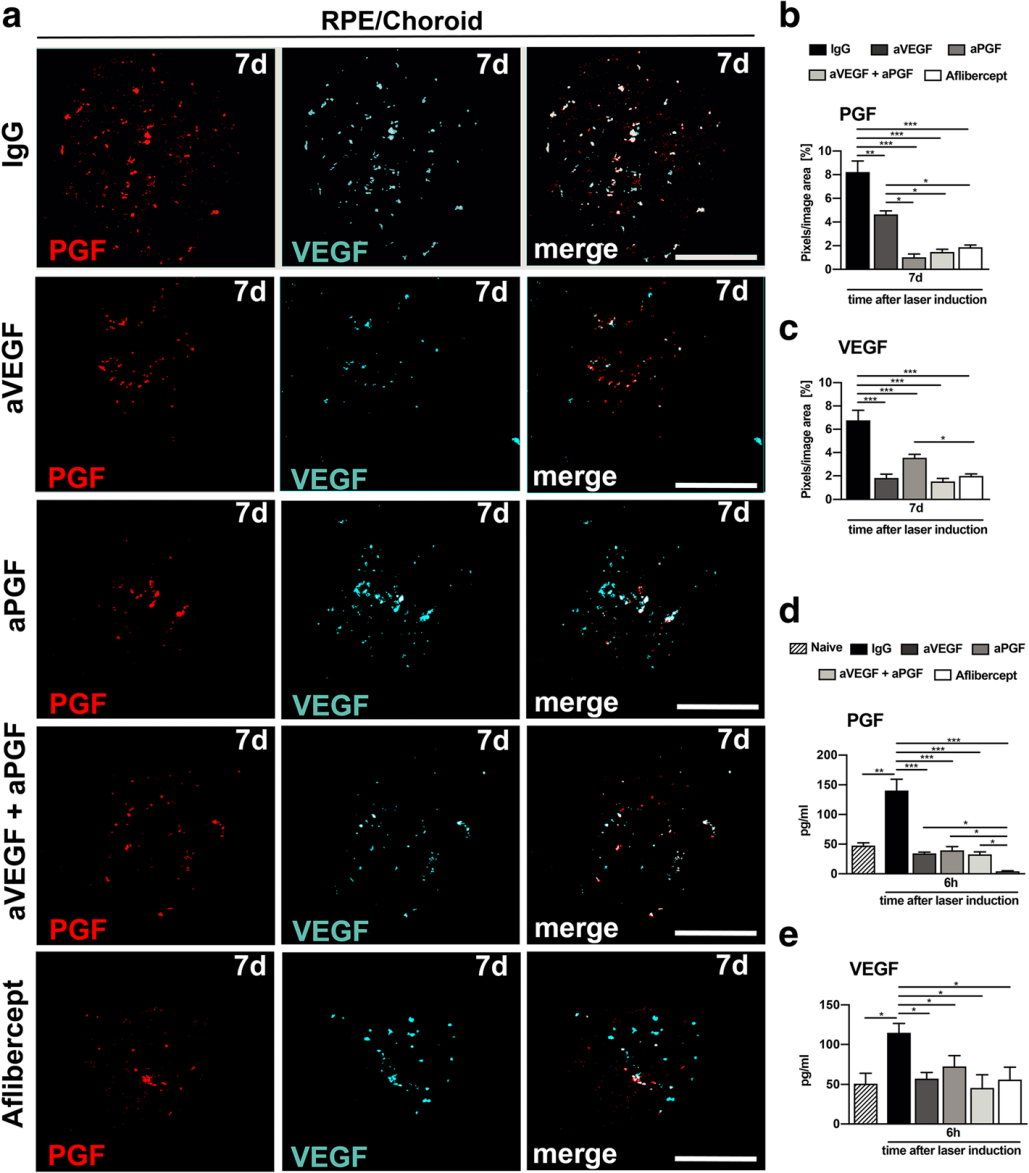
Effects of PGF and VEGF inhibition on their transcription and secretion in phagocytes. **a** Representative images of in situ hybridization (ISH) in single laser spots of RPE/choroidal flat mounts 7 days after laser damage. Probes bind specifically to mRNAs of PGF and VEGF. Scale bar: 100 μm. **b** Mean RNA expression of VEGF per laser spot in RPE/choroidal flat mounts 7 days after laser coagulation. Expression was determined by quantifying the mean colored pixels per area (*n* = 10 laser spots for IgG, *n* = 10 laser spots for aVEGF, *n* = 11 laser spots for aPGF, *n* = 10 laser spots for aVEGF/aPGF, *n* = 14 laser spots for aflibercept). **c** Mean RNA expression of PGF per laser spot in RPE/choroidal flat mounts 7 days after laser coagulation. Expression was determined by quantifying the mean colored pixels per area (*n* = 10 laser spots for IgG, *n* = 10 laser spots for aVEGF, *n* = 11 laser spots for aPGF, *n* = 10 laser spots for aVEGF/aPGF, *n* = 14 laser spots for aflibercept). **d** Cytokine levels of VEGF in RPE/choroidal flat mounts 7 days after laser damage measured by ELISA (*n* = 6 flat mounts per group). **e** Cytokine levels of PGF in RPE/choroidal flat mounts 7 days after laser damage measured by ELISA (*n* = 6 flat mounts per group). Data are shown as mean ± SEM (*P < 0.05, **P < 0.01, ***P < 0.001)

We then analyzed the effect of the different treatments on VEGF and PGF mRNA expression in phagocytes at day 7 using ISH (Fig. 6a). Intravitreal administration of aVEGF, the combination of aVEGF and aPGF as well as aflibercept strongly reduced the color pixel intensity in the laser lesions of RPE/choroidal flat mounts hybridized with VEGF probes. Treatment with aPGF also reduced pixel intensity but significantly less than the other biologics (Fig. 6b). Hybridization with PGF probes showed that all treatments attenuated PGF mRNA levels in the laser lesion with aVEGF showing the smallest effect (Fig. 6c).

Since mRNA expression levels do not unequivocally represent translation and secretion of cytokines, we measured VEGF and PGF protein levels in RPE/choroidal flat mounts using ELISAs. Both VEGF and PGF were expressed at basal levels in RPE/choroidal flat mounts of naive mice, which strongly increased 6 h after laser damage (Fig. 6d, e). Significantly reduced VEGF and PGF levels were observed in all treatment groups (Fig. 6d, e). However, PGF protein levels were fully suppressed by aflibercept only, indicating the highest efficacy of the VEGF/PGF protein trap aflibercept compared to inhibition with single or combined antibodies (Fig. 6e).

## Discussion

This study aimed to investigate the role of PGF and VEGF inhibition on neovessel formation and mononuclear phagocyte reactivity in the murine laser CNV model. Our data show that the PGF inhibition, especially with aflibercept, dampens vascular leakage and CNV 7 days after laser application. These results are in accordance with a recent report comparing antibody-mediated PGF inhibition with aflibercept at a later time point (14 days) after laser treatment [17]. In that paper, Crespo-Garcia et al. identified higher PGF and VEGF levels in the laser-damaged retina using immunostainings [17]. Here, we significantly expand these findings by showing in situ co-expression of PGF and VEGF by Iba1-positive mononuclear phagocytes in the RPE/choroid complex. These in situ hybridization data were then verified by quantitative ELISAs, again demonstrating a strong induction of VEGF and PGF protein levels in the laser CNV model and effective inhibition of both factors especially with aflibercept.

Previous reports have indicated a higher efficacy of co-inhibition of VEGF and PGF in decreasing both retinal leakage and neovascularization compared to VEGF inhibition alone [16, 17]. Our analysis of mononuclear phagocytes in both retinal and RPE/choroidal flat mounts revealed reduced microgliosis in the retina, less mononuclear phagocytes in the RPE/choroid, and significantly lower expression of PGF and VEGF after trapping PGF and VEGF. Thus, resident retinal immune cells as well as recruited macrophages seem to be both affected by intravitreal PGF/VEGF inhibition, which is in line with the proposed role for both cell populations in AMD [6]. Of note, the strongest reduction of phagocytes in laser lesions was achieved by the treatments involving PGF inactivation. This indicates that PGF seems to be important for cell recruitment and retaining them at the lesion site, which corroborates previous findings from isolated retinal immune cells [16].

The combined blockage of VEGF and PGF resulted in a more effective reduction of vascular leakage and neovascularization than both treatments alone suggesting a synergistic effect of these two compounds. Indeed, Huo et al. showed that the inhibition of PGF significantly increased the inhibitory effects of aVEGF on vessel density after laser impact [16]. In comparison to the combined therapy with aPGF and aVEGF, the administration of aflibercept showed stronger effects regarding the vascular leakage and CNV. A plausible explanation could be the fact that aflibercept binds VEGF-A not only via the amino acids necessary for VEGFR1/R2 interactions but also blocks the heparin-binding site on VEGF-A [30]. Heparin and heparan sulfate significantly contribute to the process of angiogenesis [31]. However, what remains unsolved is the question which cell types have the biggest impact on VEGF and PGF-related neovessel formation.

Pro-inflammatory cytokines are rapidly produced by mononuclear phagocytes upon activation. Here, we determined the quantitative levels of IL-6, IL-1β, and TNF in RPE/choroid samples. In contrast to previous findings derived from qPCR analyses [17], our data showed a high secretion of IL-1β in RPE/choroid samples. Since IL-1β can potently induce VEGF production by RPE cells [32], the reduced IL-1β levels in the different treatment groups may also dampen RPE-derived VEGF levels. The anti-PGF/VEGF-A treatments could possibly also limit inflammasome activation in the RPE [33, 34]. The fact that laser damage did not induce IL-1β in the retina suggests an important role of RPE cells and invading macrophages for inflammasome-dependent IL-1β secretion. As the RPE inflammasome can also trigger chemotaxis of microglia [34], anti-angiogenic therapies may indirectly modulate migration of mononuclear phagocytes. IL-6 secretion was reduced in both retinal and RPE/choroidal flat mounts after VEGF-A and PGF co-inhibition, implicating that microglia and macrophages may contribute to its secretion in both tissues. These findings are in line with a report by Levy et al., demonstrating that macrophages produce high amounts of IL-6 in apolipoprotein E-dependent conditions mimicking AMD [35].

Beside its anti-angiogenic effects, PGF blockade has recently been shown to protect other cell types in the eye. Thus, PGF inhibition by antibodies or aflibercept protected photoreceptors from light-induced degeneration [36, 37]. The modulation of microglia was not analyzed in these studies but could be a possible explanation for these positive effects of PGF inhibition on the stressed retina. Finally, aflibercept seems to have less side effects on RPE physiology than the antibody-derived molecules bevacizumab and ranibizumab [38].

Despite the findings of significantly better preclinical efficacy in reducing lesion site and immune cell activation in the laser CNV model presented here, human clinical studies showed comparable effects of aflibercept and ranibizumab in treatment-naive patients with wet AMD [39]. However, aflibercept was more effective in patients with lower baseline visual acuity, indicating a potential benefit in trapping both PGF and VEGF compared to VEGF inhibition alone [39]. Future research is needed to decipher the biological pathways affected by blockade of these two important growth factors in the retina.

## Conclusions

In summary, this study for the first time showed that PGF inhibition, most effective as trap using aflibercept, reduced phagocyte-related mRNA expression and secretion of VEGF-A and PGF as well as pro-inflammatory cytokines in the laser CNV model mimicking wet AMD. Aflibercept showed the highest efficacy in preventing vascular leakage and CNV. Pharmacological targeting of pro-angiogenic and pro-inflammatory pathways simultaneously may therefore provide a novel approach for the treatment of neovascular AMD.

## Additional files

Additional file 1: **Figure S1.** Aflibercept does not attenuate wound healing. a Top, representative mouse fundus images analyzed with the Heidelberg Spectralis IR-mode at days 0, 3, and 7 after laser coagulation. Bottom, representative cross-section images of laser lesion sites. b Quantification of laser spot size at day 0 (*n* = 24 eyes for IgG, *n* = 22 eyes for aVEGF, *n* = 24 eyes for aPGF, *n* = 20 eyes for aVEGF/aPGF, *n* = 22 eyes for aflibercept). c Quantification of laser spot size at day 3 (*n* = 22 eyes for IgG, *n* = 15 eyes for aVEGF, *n* = 16 eyes for aPGF, *n* = 15 eyes for aVEGF/aPGF, *n* = 18 eyes for aflibercept). d Quantification of laser spot size at day 7 (*n* = 15 eyes for IgG, *n* = 17 eyes for aVEGF, *n* = 19 eyes for aPGF, *n* = 15 eyes for aVEGF/aPGF, *n* = 20 eyes for aflibercept). Data are shown as mean ± SEM. (JPG 2535 kb)

Additional file 2: **Figure S2.** Aflibercept does not affect mononuclear phagocyte morphology. a Quantification of immune cell morphology in laser spots 3 days after laser coagulation in retinal flat mounts (*n* = 11 laser spots per group). b Quantification of immune cell morphology in laser spots 7 days after laser coagulation in retinal flat mounts (*n* = 11 laser spots per group). c Quantification of immune cell morphology in laser spots 3 days after laser coagulation in RPE/choroidal flat mounts (*n* = 11 laser spots per group). d Quantification of immune cell morphology in laser spots 7 days after laser coagulation in RPE/choroidal flat mounts (*n* = 11 laser spots per group). All images were analyzed using a grid image analysis system (ImageJ). (JPG 1098 kb)

## Abbreviations

Aflib.: Aflibercept
AMD: Age-related macular degeneration
aPGF: Anti-placental growth factor
aVEGF: Anti-vascular endothelial growth factor
CNV: Choroidal neovascularization
IgG: Immunoglobulin G
IHC: Immunohistochemistry
IL-1β: Interleukin-1β
IL-6: Interleukin-6
ISH: In situ hybridization
NBF: Neutral buffered formalin
OCT: Optical coherence tomography
PBS: Phosphate-buffered saline
PGF: Placental growth factor
ROI: Region of interest
RPE: Retinal pigment epithelium
TNF: Tumor necrosis factor
VEGF: Vascular endothelial growth factor
VEGFR1: Vascular endothelial growth factor Receptor 1
