# Supplementary figures and images for: Co-inhibition of PGF and VEGF blocks their expression in mononuclear phagocytes and limits neovascularization and leakage in the murine retina
Source: J Neuroinflammation. 2019 Feb 7;16:26. doi: 10.1186/s12974-019-1419-2 (PMC6366121; doi:10.1186/s12974-019-1419-2)

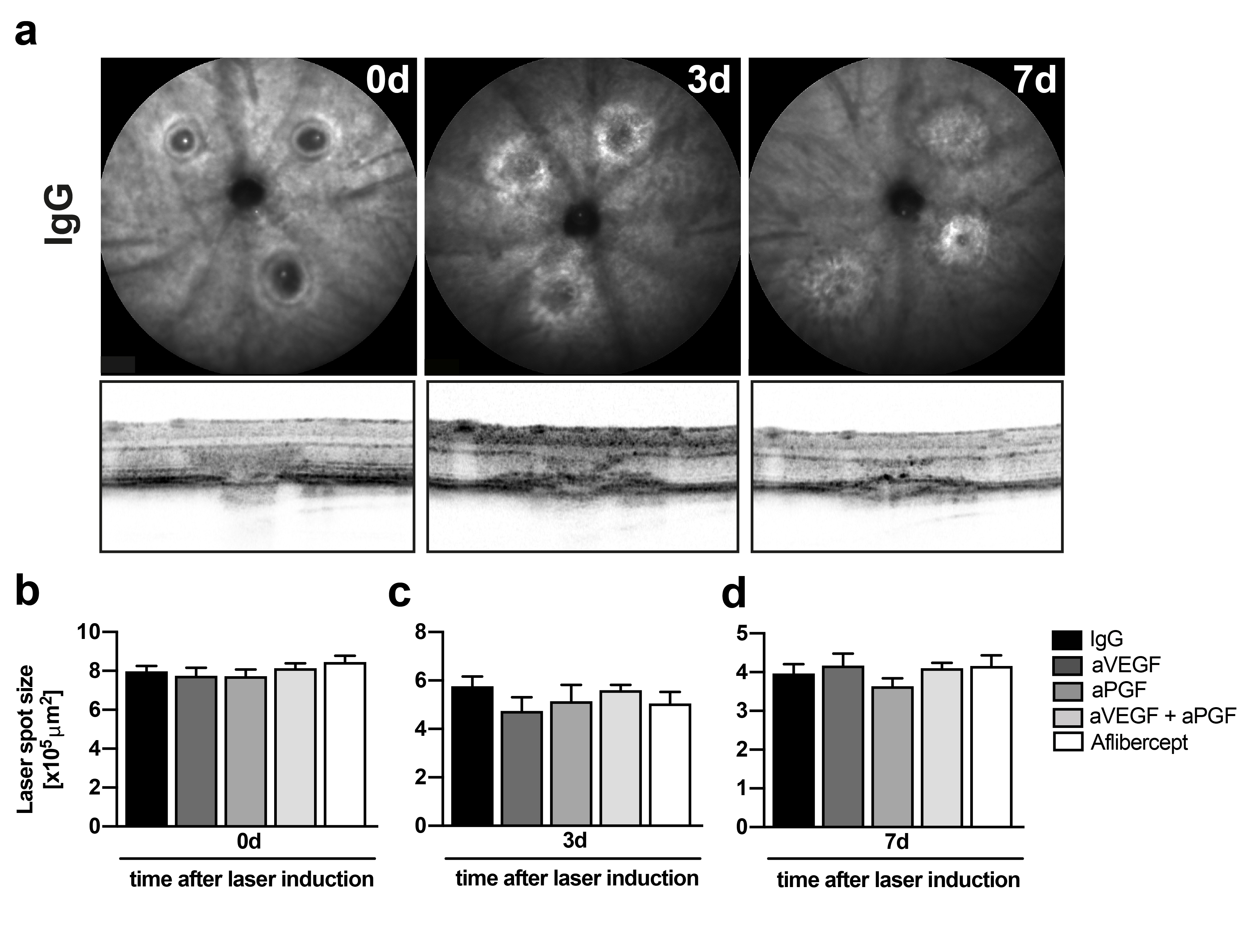

Supplement: Supplementary file 1 — Figure S1. Aflibercept does not attenuate wound healing. a Top, representative mouse fundus images analyzed with the Heidelberg Spectralis IR-mode at days 0, 3, and 7 after laser coagulation. Bottom, representative cross-section images of laser lesion sites. b Quantification of laser spot size at day 0 (n = 24 eyes for IgG, n = 22 eyes for aVEGF, n = 24 eyes for aPGF, n = 20 eyes for aVEGF/aPGF, n = 22 eyes for aflibercept). c Quantification of laser spot size at day 3 (n = 22 eyes for IgG, n = 15 eyes for aVEGF, n = 16 eyes for aPGF, n = 15 eyes for aVEGF/aPGF, n = 18 eyes for aflibercept). d Quantification of laser spot size at day 7 (n = 15 eyes for IgG, n = 17 eyes for aVEGF, n = 19 eyes for aPGF, n = 15 eyes for aVEGF/aPGF, n = 20 eyes for aflibercept). Data are shown as mean ± SEM. (JPG 2535 kb) [file 12974_2019_1419_MOESM1_ESM.jpg]

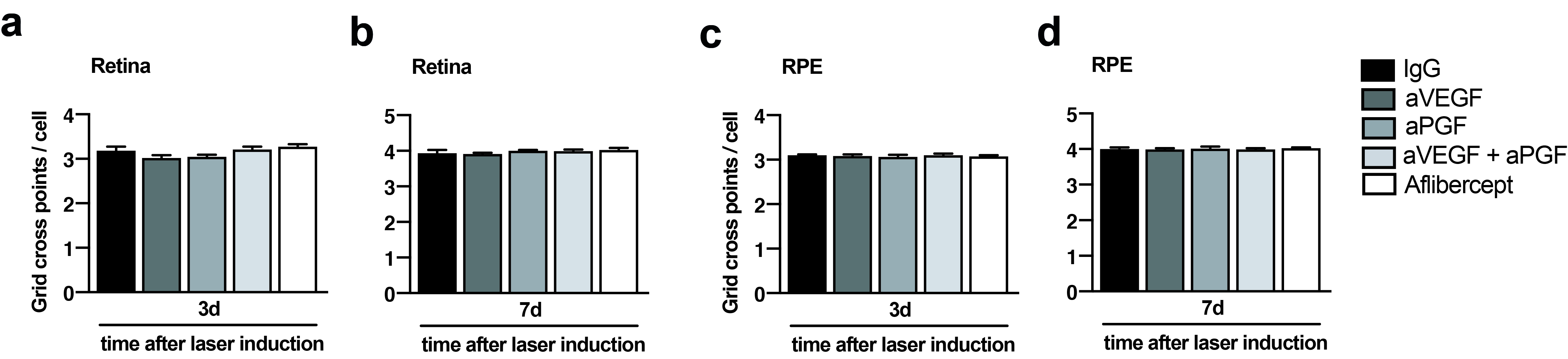

Supplement: Supplementary file 2 — Figure S2. Aflibercept does not affect mononuclear phagocyte morphology. a Quantification of immune cell morphology in laser spots 3 days after laser coagulation in retinal flat mounts (n = 11 laser spots per group). b Quantification of immune cell morphology in laser spots 7 days after laser coagulation in retinal flat mounts (n = 11 laser spots per group). c Quantification of immune cell morphology in laser spots 3 days after laser coagulation in RPE/choroidal flat mounts (n = 11 laser spots per group). d Quantification of immune cell morphology in laser spots 7 days after laser coagulation in RPE/choroidal flat mounts (n = 11 laser spots per group). All images were analyzed using a grid image analysis system (ImageJ). (JPG 1098 kb) [file 12974_2019_1419_MOESM2_ESM.jpg]
